# SMARCA4 promotes lineage plasticity and enzalutamide resistance in prostate cancer by regulating PROX1 via H3K27 acetylation

**DOI:** 10.1038/s41420-026-03068-0

**Published:** 2026-03-25

**Authors:** Chenwei Wu, Mayao Luo, Chaojian Wu, Yi Yuan, Yadong Li, Yuanpeng Liao, Yifan Zhang, Xin Huang, Mengqi Wang, Shidong Lv, Qiang Wei

**Affiliations:** 1 Department of Urology, Nanfang Hospital, Southern Medical University, Guangzhou, Guangdong China; 2 Department of Urology, Guangdong Cardiovascular Institute, Guangdong Provincial People’s Hospital, Guangdong Academy of Medical Sciences, Southern Medical University, Guangzhou, Guangdong China; 3 Department of Urology, Ganzhou Hospital-Nanfang Hospital, Southern Medical University, Ganzhou, Jiangxi China

**Keywords:** Prostate cancer, Drug development

## Abstract

Enzalutamide resistance is a dynamic process often culminating in the aggressive progression to neuroendocrine prostate cancer (NEPC). This lineage plasticity is hypothesized to be driven by underlying epigenetic alterations, yet the core molecular drivers remain unclear. Elucidating these factors is of significant clinical importance for overcoming resistance. To model this transition, we established a dynamic gradient-resistant cell model simulating the clinical response to enzalutamide, and found robust upregulation of the chromatin remodeling factor SMARCA4 in resistant cells. Both in vitro and in vivo experimental results demonstrated that inhibiting SMARCA4 effectively suppresses tumor progression and reverses neuroendocrine transformation. Mechanistically, integrated multi-omics analysis, correlation studies, and protein interaction experiments revealed the transcription factor PROX1 as a crucial downstream target of SMARCA4, where its inhibition alone was sufficient to reverse the aggressive malignancy and neuroendocrine characteristics of resistant cells. We further demonstrated that SMARCA4 enhances H3K27ac levels and chromatin accessibility at the PROX1 locus to regulate its expression. Importantly, the tumor-suppressive effect of SMARCA4 knockdown could be rescued by histone deacetylase inhibitors (HDACi), achieving a level of recovery comparable to PROX1 overexpression. In summary, this study defines a core epigenetic pathway, showing that increased SMARCA4 activity promotes luminal-to-neuroendocrine transformation by enhancing histone acetylation and chromatin accessibility at the PROX1 locus. Targeting the SMARCA4-PROX1 axis provides a valuable therapeutic strategy for combating enzalutamide resistance and NEPC progression.

## Introduction

Prostate cancer (PCa) is classically recognized as an androgen-driven malignancy, and standard therapeutic strategies rely on blocking the interaction between androgens and the androgen receptor (AR) [1]. However, a significant clinical challenge is the development of acquired resistance following androgen deprivation therapy (ADT). These resistant tumors often undergo a profound lineage switch, characterized by independence from the classical AR signaling pathway and the high expression of neuroendocrine markers. This progression ultimately results in the highly aggressive variant, neuroendocrine prostate cancer (NEPC) [2, 3]. Due to its inherent aggressiveness and poor response to subsequent treatments, NEPC currently lacks effective therapeutic options in clinical practice [4].

Tumor progression to a drug-resistant stage is frequently accompanied by lineage plasticity [5]. As the disease advances, prostate cancer undergoes dynamic lineage alterations, with current research heavily focused on the phenomenon of neuroendocrine transformation. Epigenetic regulation is recognized as a core mechanism enabling tumor cells to undergo lineage plasticity, thereby facilitating cellular adaptation and survival under therapeutic pressure [6].

Current research indicates that neuroendocrine transformation (NET) is accompanied by significant dynamics in chromatin accessibility and transcriptional reprogramming [7]. Numerous transcription factors (TFs) play crucial roles in this process of lineage plasticity following therapeutic intervention. For instance, the histone methyltransferase EZH2 promotes NET by suppressing tumor suppressor RB1 and AR signaling via histone modification, and its inhibitors can delay NEPC progression. Similarly, the histone demethylase LSD1 activates neuroendocrine genes through histone modification [8], and DNA methyltransferases accelerate NET by silencing AR pathway genes through DNA methylation [9]. However, clinical trials targeting these specific epigenetic regulators have shown limited efficacy [10]. Furthermore, current research has predominantly focused on the static effects of DNA methylation and histone modifications on NET, often overlooking the critical role of dynamic changes in three-dimensional chromatin structure in driving this transformation.

Utilizing a model where enzalutamide concentration was gradually increased, we successfully established enzalutamide-resistant cells at various stages. Our analysis of these cells revealed diverse epigenetic regulatory changes, notably dynamic alterations in chromatin accessibility. Central to this process is SMARCA4, a key catalytic subunit of the SWI/SNF chromatin remodeling complex, which functions by remodeling chromatin structure through ATP hydrolysis to regulate gene transcription. The role of SMARCA4 in cancer is complex: mutations or deficiencies promote progression and drug resistance in cancers such as lung and ovarian cancer [11–13]. Conversely, its high expression in gliomas correlates with proliferation and invasion, where its knockdown inhibits tumor growth and enhances chemotherapy efficacy. In prostate cancer, while SMARCA4 maintains androgen receptor (AR) signaling in the hormone-sensitive phase [14], its expression is significantly elevated in aggressive neuroendocrine prostate cancer (NEPC) tissues [15]. However, the precise role of SMARCA4 in driving NEPC progression remains to be fully elucidated.

Given that SMARCA4 typically requires cooperative action with other transcription factors or chromatin remodeling factors to fully exert its function in gene expression regulation, we sought to identify its key cooperating partner. Through in-depth multi-omics analysis, we discovered that the transcription factor PROX1 exhibits a high correlation with SMARCA4-mediated chromatin remodeling in resistant cells. PROX1 (Prospero Homeobox 1) is an evolutionarily conserved homeobox transcription factor crucial for core regulatory roles in embryonic development (including neurogenesis and lymphatic formation) and adult cell fate determination [16, 17]. Furthermore, PROX1 is recognized as a key transcription factor involved in the progression of various cancers [18].

H3K27ac is a critical histone modification marking active enhancers and promoters, which functions cooperatively with the acetyltransferases p300/CBP to exert its regulatory effect. Mechanistically, SMARCA4 facilitates p300-mediated H3K27ac modification and drives the expression of targets like the proto-oncogene MYC [19, 20]. Consistent with this mechanism, our results revealed a significant increase in H3K27ac enrichment specifically at the PROX1 promoter region in resistant cells, while H3K4me1 and H3K27me3 modifications remained unchanged. A series of in vitro functional experiments further suggests that SMARCA4 regulates PROX1 activation through the modulation of H3K27ac modification.

In this study, we established a gradient-resistant cell model and discovered that enzalutamide-resistant cells exhibit significantly enhanced chromatin accessibility, with SMARCA4 playing a critical role in this change. Our results demonstrate that SMARCA4 facilitates histone acetylation at the PROX1 locus, mediates PROX1 chromatin remodeling, and thereby drives neuroendocrine transformation in prostate cancer.

## Results

### Lineage plasticity as a key regulator of enzalutamide resistance in prostate cancer

**Fig. 1:**
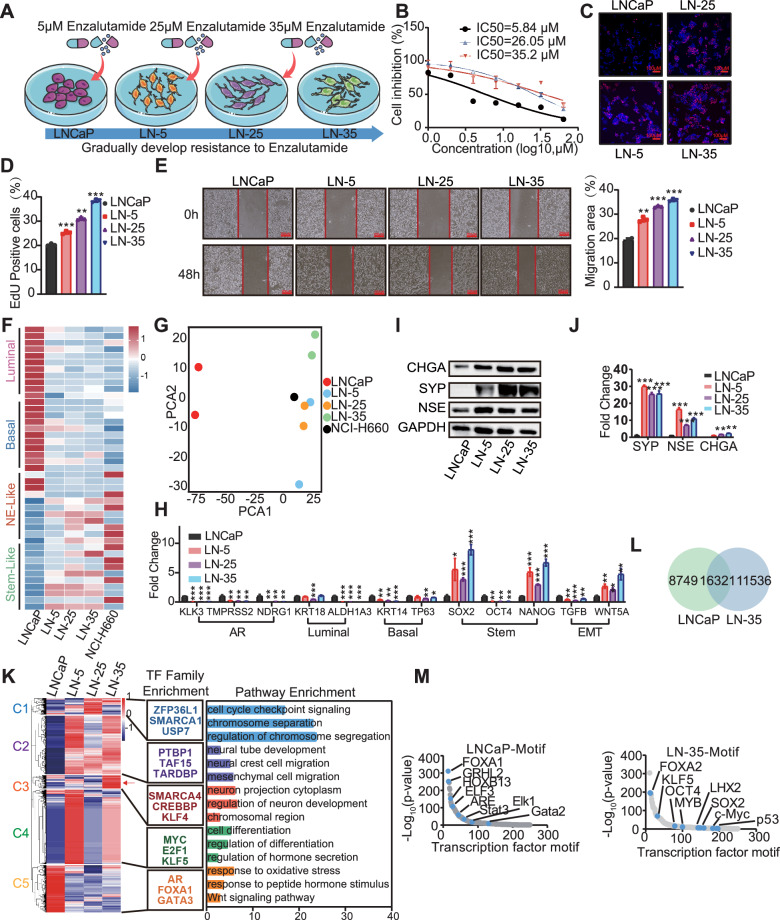
Transcriptomic reprogramming and lineage plasticity during enzalutamide resistance. **A** Schematic representation of the stepwise establishment of enzalutamide-resistant LNCaP sublines (LN-5, LN-25, and LN-35) via increasing drug concentrations (5–35 μM). **B** Evaluation of drug resistance. Calculated IC₅₀ values were assessed for each resistant cell line using the CCK-8 assay after 7 days of exposure to increasing concentrations of enzalutamide. **C** Representative fluorescence images of EdU staining. Proliferating cells are visualized in red (EdU), and nuclei are counterstained with DAPI (blue). **D** Quantitative analysis of the percentage of EdU-positive cells from the experiment shown in (**C**). **E** Evaluation of cell migration via wound healing assay. Representative images of LNCaP and resistant sublines were captured at 0 and 48 hours. The statistical quantification of the wound closure rate is shown in the adjacent graph. **F** Heatmap comparison of lineage gene signatures across LNCaP, enzalutamide-resistant sublines, and the NEPC reference cell line NCI-H660. **G** PCA plot of the transcriptomes from LNCaP, resistant cells, and NCI-H660 cells. **H** mRNA expression levels of representative genes associated with key signaling pathways in LNCaP and resistant sublines. Expression of neuroendocrine (NE) markers at the protein (**I**) and mRNA (**J**) levels in LNCaP and resistant sublines. **K** Integrated transcriptomic analysis of LNCaP and resistant sublines. Left panel: unsupervised clustering analysis. Middle panel: enrichment analysis of transcription factor families for each gene cluster. Right panel: pathway enrichment analysis for each gene cluster. **L** Venn diagram of ATAC-seq peaks unique and common to LNCaP and LN-35 cells. **M** Ranked motif of accessible chromatin regions specific to LNCaP and LN-35 cells. (*p < 0.05, **p < 0.01, ***p < 0.001).

To investigate the mechanisms underlying enzalutamide resistance, we established a panel of enzalutamide-resistant cell lines derived from LNCaP cells, designated as LN-5, LN-25, and LN-35 (Fig. 1A). The drug-resistant phenotype was validated by IC50 measurements, which confirmed that the resistant sublines exhibited significant tolerance to the target enzalutamide concentrations (Fig. 1B). We then sought to assess the change in malignant phenotype associated with resistance. The CCK-8 and EdU assays demonstrated that the resistance cells possessed enhanced proliferative capacity relative to the LNCaP cells (Fig. 1C, D, and Supplementary Fig. 1A). Furthermore, both the wound healing and Transwell assays showed that the resistant sublines acquired significantly enhanced migratory and invasive capabilities (Fig. 1E and Supplementary Fig. 1B).

To comprehensively capture the transcriptomic alterations during the development of enzalutamide resistance, we compared the expression of canonical lineage markers. We observed a trend where resistant cells exhibited a downward expression of luminal markers and an upregulation of neuroendocrine-like and stem-like signaling markers. Based on the GSE138460 dataset, this expression profile closely aligned with that of the NCI-H660 cell line [21] (Fig. 1F). Further supporting this lineage switch, Principal Component Analysis (PCA) showed that the resistant cells clustered in close to NCI-H660 cells, strongly suggesting neuroendocrine transdifferentiation (NETD) (Fig. 1G). Gene Set Enrichment Analysis (GSEA) of the most resistant LN-35 cells further demonstrated significant enrichment in pathways related to stem-like signaling (Supplementary Fig. 1C). Subsequent validation across the resistant cell lines confirmed these shifts, revealing decreased mRNA expression of the luminal signaling pathways, concurrent with elevated expression of both stemness and neuroendocrine (NE) signaling markers (Fig. 1H–J). To delineate the transcriptional dynamics during enzalutamide resistance development, we performed K-means clustering analysis to categorize the differentially expressed genes (DEGs) across the resistant cells. For each cluster, we conducted transcription factor family and motif enrichment analyses to elucidate the underlying biological mechanisms (Fig. 1K). While the enriched transcription factors and pathways varied across resistance stages, a commonly enriched pathway across all resistant cell lines (C2) suggested that neural-related alterations participate throughout the entire resistance process, in particular, it exhibits more pronounced neuroendocrine features in LN-35 cells. Furthermore, the highly resistant LN-35 cells (C3) showed specific enrichment for transcription factors involved in chromatin remodeling. Changes in chromatin accessibility further validated these conclusions, as evident differences were observed between the LNCaP and LN-35 cells (Fig. 1L, Supplementary Fig. 1D). Motif and pathway enrichment analyses of peaks unique to each cell type revealed that LNCaP-specific peaks were primarily associated with the AR signaling pathway, whereas those unique to LN-35 cells were mainly linked to the Neuroendocrine (NE) signaling pathway (Fig. 1M, Supplementary Fig. 1E). Collectively, these findings demonstrate that our engineered resistant cells underwent lineage plasticity during drug resistance acquisition, with the LN-35 subline exhibiting a clear transition driven by chromatin remodeling and neuroendocrine transdifferentiation.

### Chromatin remodeling and transcriptional reprogramming are coordinated and dynamically orchestrated during the acquisition of enzalutamide resistance

**Fig. 2:**
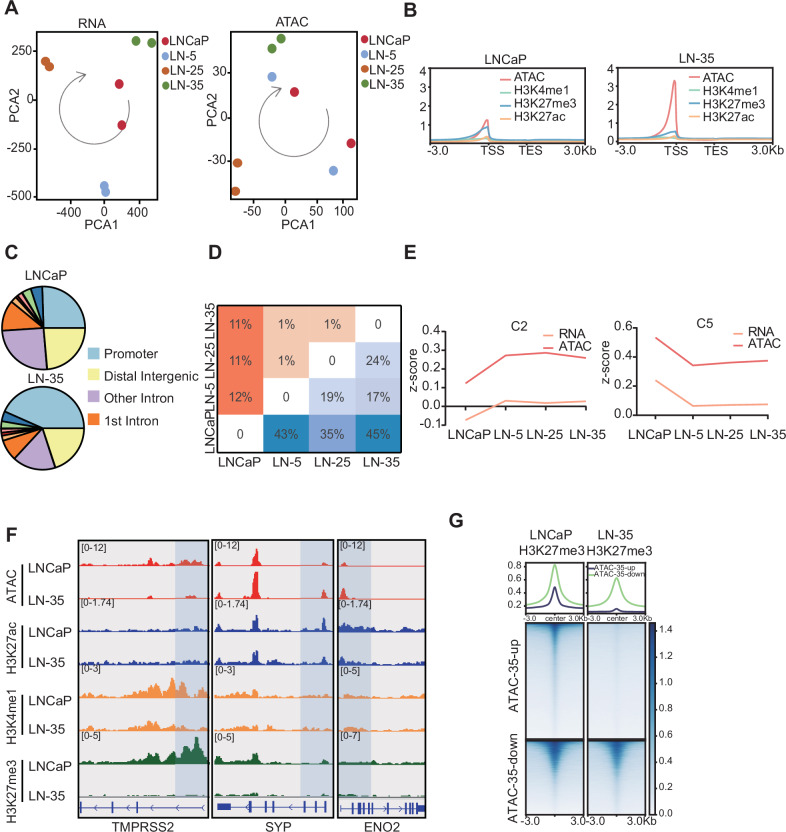
Coordinated epigenomic and transcriptomic remodeling during enzalutamide resistance. **A** PCA of RNA-seq and ATAC-seq data from LNCaP and resistant cells. **B** Aggregate profiles of histone modifications and chromatin accessibility across gene bodies (from TSS to TES) in LNCaP and LN-35 cells. **C** Genomic distribution of ATAC-seq peaks across different functional regions. **D** Numbers of differentially expressed genes and differentially accessible peaks in resistant cell lines compared to parental LNCaP cells. **E** Correlation analysis between transcriptomic changes and alterations in chromatin accessibility. **F** Chromatin accessibility track and histone modification signals track at the genomic loci of *TMPRSS2* and NE genes. **G** Heatmap of H3K27me3 in LNCaP versus LN-35 cells.

Our transcriptomic and chromatin profiling data captured the continuum of enzalutamide resistance development (Fig. 2A). Compared to the LNCaP cells, LN-35 subline exhibited significant alterations in chromatin accessibility, with a notable enrichment of these changes occurring in promoter regions—a process potentially mediated by dynamic histone modifications (Fig. 2B, C). Throughout the different stages of resistance, we identified a large number of differentially expressed genes (DEGs) and differentially accessible peaks (DAPs) between each resistant cell line and the LNCaP cells (Fig. 2D). We calculated the Pearson correlation coefficients between the transcriptional and chromatin patterns of each cluster to determine if these alterations follow coordinated regulatory trends. The results revealed a strong positive correlation between the dynamic changes in chromatin accessibility and the corresponding transcriptional changes across all resistance stages (Fig. 2E, Supplementary Fig. 2A). Our previous results demonstrated lineage plasticity in the resistant cells, characterized by the downregulation of AR signaling and upregulation of NE signaling. We therefore sought to determine whether chromatin accessibility peaks at the Transcription Start Sites (TSSs) of relevant signaling pathways exhibited corresponding alterations. Our data revealed a decreasing chromatin accessibility at the TMPRSS2 in LN-35 cells, concurrent with increasing trends at the SYP and ENO2 TSSs (Fig. 2F). This finding further supported the dynamic coordination between chromatin alterations and transcriptomic changes. Given that alterations in chromatin accessibility may be mediated by changes in histone modifications, we subsequently analyzed the CUT&Tag data for three common histone modifications. We observed that none of the three common histone modifications investigated perfectly aligned with the alterations in the AR and NE axes (Fig. 2F). Further comparison of the heatmaps revealed that the H3K27me3 profile showed the strongest correlation with trends in chromatin accessibility, characterized by a significant depletion of H3K27me3 in the open chromatin regions of LN-35 cells (Fig. 2G, Supplementary Fig. 2B). To investigate the global changes of H3K27me3, we analyzed the differences in its binding peaks. The heatmap results showed that LN-35 cells have fewer binding peaks compared to LNCaP cells (Supplementary Fig. 2C). Western blotting confirmed this finding, showing decrease in H3K27me3 protein levels in LN-35 cells compared to LNCaP cells (Supplementary Fig. 2D). Collectively, these results indicate that during the development of enzalutamide resistance, changes in chromatin accessibility are dynamically orchestrated with transcriptional reprogramming. This critical process may be regulated by multiple factors, but is primarily mediated by alterations in H3K27me3 modification.

### Aberrant SMARCA4 activation facilitates prostate cancer malignancy

**Fig. 3:**
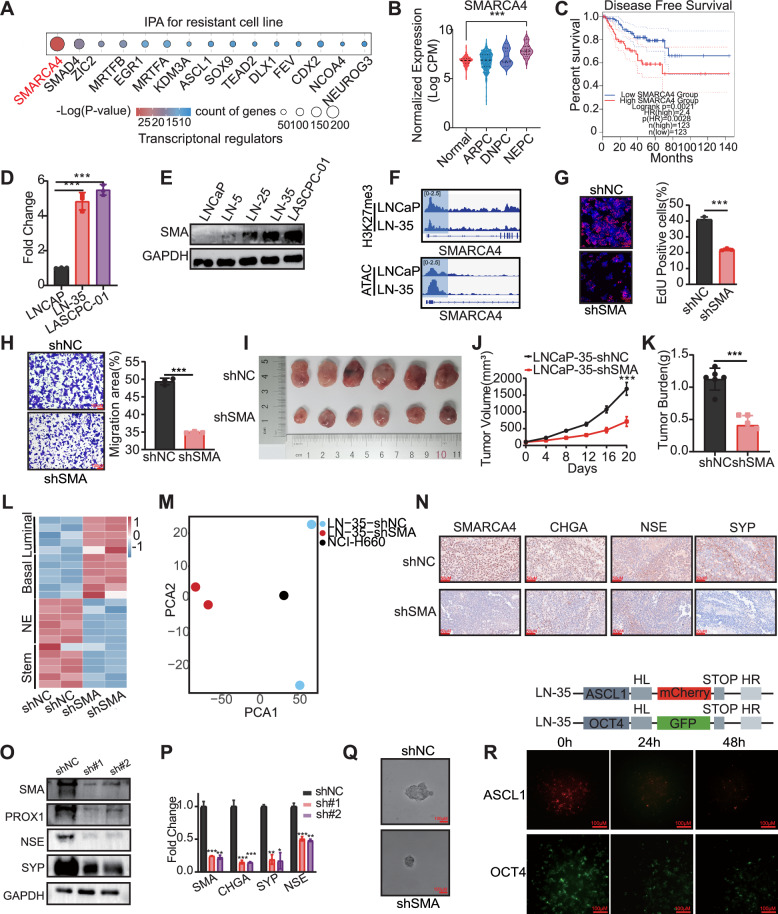
Aberrant SMARCA4 activation drives prostate cancer progression and neuroendocrine differentiation. **A** Results of IPA based on the transcriptome of LN-35 cells. **B** SMARCA4 expression across human prostate cancer subtypes (Data source: The Human Protein Atlas). **C** DFS analysis of prostate cancer patients in the TCGA cohort stratified by SMARCA4 expression. SMARCA4 expression at the mRNA (**D**) and protein (**E**) levels in LNCaP, resistant cells, and LASCPC-01 cells. **F** Chromatin accessibility and H3K27me3 enrichment at the SMARCA4 TSS in LNCaP versus LN-35 cells. **G** Representative EdU staining images (left) and quantification (right) of LN-35 cells following SMARCA4 knockdown. Nuclei were counterstained with DAPI (blue), and proliferating cells were labeled with EdU (red). **H** Representative images (left) and quantification (right) of Transwell assay in SMARCA4-knockdown LN-35 cells. Representative images of xenograft tumors (**I**), tumor growth curves (**J**), and final tumor weights (**K**) derived from LN-35 cells expressing control (shNC) or SMARCA4-targeting (shSMARCA4) shRNAs (*n* = 6 per group). **L** Heatmap of differentially expressed signaling pathway genes in LNCaP-35 cells after SMARCA4 knockdown. **M** Principal component analysis (PCA) of transcriptomic profiles from LN-35, SMARCA4-knockdown LN-35, and NCI-H660 cells. **N** Immunohistochemical (IHC) staining of neuroendocrine markers in xenograft tumors derived from shNC and shSMARCA4 groups harvested at day 20. Expression levels of neuroendocrine markers (Protein **O**, mRNA **P**) in xenograft tumors from shNC and shSMARCA4 groups. **Q** Representative images of tumor sphere formation in control versus SMARCA4-knockdown LN-35 cells. **R** Time-course monitoring of fluorescence intensity for ASCL1 (red) and OCT4 (green) knock-in reporters in LN-35 cells following SMARCA4 knockdown. (*p < 0.05, **p < 0.01, ***p < 0.001).

To investigate the molecular drivers of enzalutamide resistance, we performed Ingenuity Pathway Analysis (IPA) on our transcriptomic data. The results strongly indicated that the chromatin remodeling factor SMARCA4 plays a critical role in the resistance process (Fig. 3A). This finding was consistent with our prior analysis, which identified SMARCA4 as a key enriched transcription factor in the LN-35 subline (Fig. 1K). To establish the clinical relevance of SMARCA4, we analyzed public databases. Data from The Human Protein Atlas (HPA) revealed a higher expression level of SMARCA4 in NEPC compared to normal tissue (Fig. 3B). Furthermore, analysis of The Cancer Genome Atlas (TCGA) data demonstrated that high SMARCA4 expression was significantly associated with a worse prognosis in prostate cancer patients (Fig. 3C). We subsequently validated these findings in our cellular models. Both mRNA and protein levels of SMARCA4 were found to be markedly elevated in the LN-35 and LASCPC-01 cells when compared to the LNCaP cells (Fig. 3D, E). Furthermore, we observed increased chromatin accessibility concurrent with a decrease in H3K27me3 peaks in LN-35 cells relative to LNCaP cells (Fig. 3F). These results are consistent with our prior data and collectively suggest that the activation of SMARCA4 may be a key factor promoting enzalutamide resistance. To confirm its functional significance in malignant progression, we utilized SMARCA4 knockdown, which resulted in a suppression of key oncogenic phenotypes. EdU and CCK-8 assays revealed significantly reduced proliferation (Fig. 3G, Supplementary Fig. 3A), while Transwell and wound healing assays evidenced impaired cellular migration and invasion (Fig. 3H, Supplementary Fig. 3B). Furthermore, recognizing the frequent association between neuroendocrine progression and anti-apoptotic activity [22], flow cytometric analysis confirmed that SMARCA4 knockdown increased the proportion of apoptotic LN-35 cells (Supplementary Fig. 3C). Crucially, these findings translated into a therapeutic effect in vivo, where SMARCA4 knockdown led to a significant reduction in tumor volume in mouse models compared to controls (Fig. 3I–K).

To elucidate the potential role of SMARCA4 in Neuroendocrine Transdifferentiation, we initially utilized data from GSE77930 to analyze the correlation between SMARCA4 mRNA expression and that of classical neuroendocrine markers [6]. This analysis revealed a robust positive correlation, suggesting a coordinated regulatory relationship. Furthermore, single-cell RNA sequencing data from GSE264573 and GSE246155, derived from human NEPC samples, corroborated these findings by demonstrating significant enrichment of SMARCA4 and classical neuroendocrine markers in NEPC [23, 24].(Supplementary Fig. 3D–G). Subsequently, we observed SMARCA4 knockdown in LN-35 cells resulted in a significant shift in lineage marker expression: markers associated with Luminal and Basal signaling were increased, while markers characteristic of NE-Like and Stem-Like signaling were decreased (Fig. 3L). The magnitude of this lineage reversal was further highlighted by Principal Component Analysis (PCA), which showed a distinct separation between SMARCA4-knockdown LN-35 cells and NCI-H660 cells (Fig. 3M). The pivotal role of SMARCA4 in driving the NE phenotype was definitively validated through both in vitro and in vivo functional assays. Upon SMARCA4 knockdown, we observed a consistent decreasing trend in both the mRNA and protein levels of classical neuroendocrine (NE) markers (Fig. 3O, P, Supplementary Fig. 3H, I). These results were further confirmed by Immunohistochemistry (IHC), which revealed a significant reduction in the expression of key NE markers in the tumors following SMARCA4 knockdown (Fig. 3N). Additionally, given the close link between NE and stemness, we assessed the cellular stemness capacity. The sphere formation assay demonstrated a significant reduction in the sphere-forming ability of the drug-resistant cells after SMARCA4 knockdown (Fig. 3Q, Supplementary Fig. 3J). To dynamically track the impact of SMARCA4 on lineage plasticity, we engineered LN-35 cells by introducing fluorescent reporters upstream of the terminators for ASCL1 and OCT4. Following SMARCA4 silencing, we observed a gradual decrease in the corresponding fluorescence intensity over time (Fig. 3R). In parallel, pathway enrichment analysis of the transcriptome upregulated following SMARCA4 knockdown revealed a significant enrichment of multiple pathways related to DNA damage repair (DDR) (Supplementary Fig. 3K). This suggests that the pro-survival functions of SMARCA4 extend to regulating genome integrity pathways. In summary, chromatin remodeler SMARCA4 as a critical driver underlying both malignant progression and neuroendocrine transformation in enzalutamide-resistant prostate cancer cells.

### SMARCA4 drives neuroendocrine transformation through upregulating PROX1

**Fig. 4:**
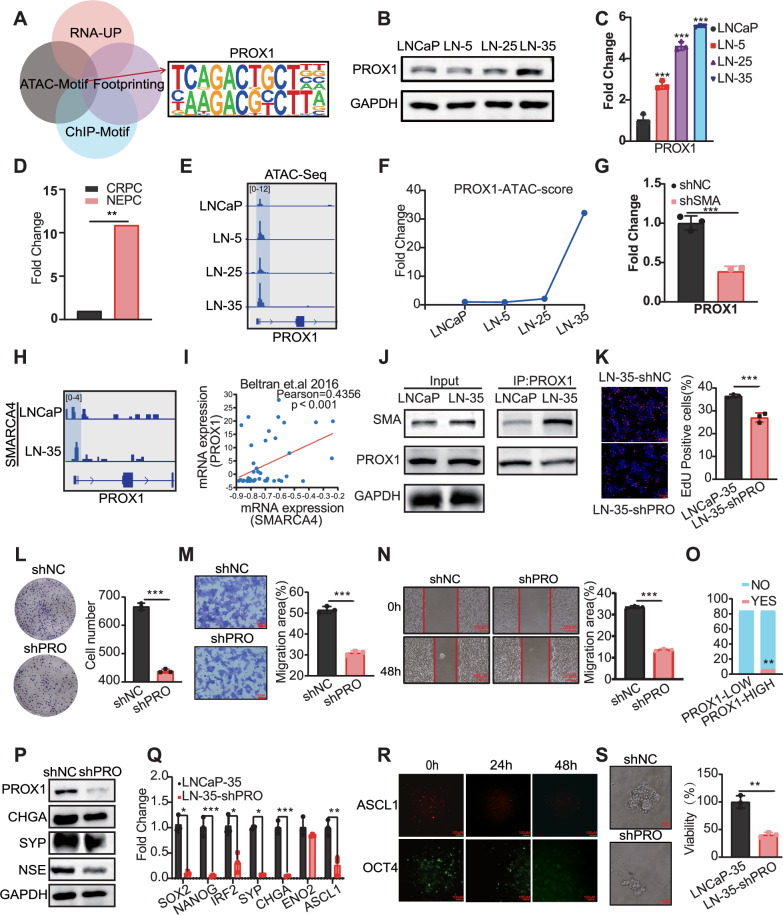
SMARCA4 promotes increased chromatin accessibility at the PROX1 locus. **A** Integrated multi-omics analysis reveals potential downstream targets of SMARCA4. Protein (**B**) and mRNA (**C**) expression levels of PROX1 in LNCaP and resistant cells. **D** Comparison of PROX1 mRNA expression between CRPC and NEPC in the GSE138460 dataset. **E** Chromatin accessibility at the PROX1 TSS in LNCaP versus resistant cells. **F** Transcription factor footprinting scores for PROX1 derived from ATAC-seq data. **G** PROX1 mRNA expression in LN-35 cells following SMARCA4 knockdown. **H** Representative ChIP-seq tracks showing SMARCA4 binding at the PROX1 tss in LNCaP versus LN-35 cells. **I** Correlation analysis between SMARCA4 and PROX1 mRNA expression in the GSE77930 dataset. **J** Protein interaction between SMARCA4 and PROX1 detected by CO-IP. **K** Representative EdU staining images (left) and quantification (right) of LNCaP-35 cells following PROX1 knockdown. Nuclei were counterstained with DAPI (blue), and proliferating cells were labeled with EdU (red). **L** Representative images and quantification of colony formation in LN-35 cells following PROX1 knockdown, cultured for 10 days. **M** Representative images of Transwell assay in PROX1-knockdown LN-35 cells and quantification of the relative number of migrated cells normalized to the control group (set as 100%). **N** Wound healing assay in LN-35 cells after PROX1 knockdown, showing representative images at 0 and 48 hours and quantification of wound closure rate. **O** Proportion of patients exhibiting neuroendocrine (NE) features in the SU2C cohort, stratified by high versus low PROX1 expression. Protein (**P**) and mRNA (**Q**) expression levels of neuroendocrine markers in LNCaP-35 cells following PROX1 knockdown. **R** Time-course monitoring of ASCL1 (red) and OCT4 (green) reporter fluorescence in LN-35 cells following PROX1 knockdown over 48 hours. **S** Representative images and quantification of tumor sphere size in control versus PROX1-knockdown LN-35 cells. (*p < 0.05, **p < 0.01, ***p < 0.001).

To mechanistically connect SMARCA4 to the observed phenotypic changes, we performed an integrated analysis combining our RNA-sequencing (upregulated genes), ATAC-sequencing (motif and footprinting analyses), and the motif of SMARCA4 ChIP-sequencing. This integrative approach collectively identified PROX1 as a compelling potential downstream target (Fig. 4A). Subsequent validation experiments confirmed that PROX1 is progressively upregulated in LN-35 cells, showing increased protein and mRNA expression levels compared to parental cells (Fig. 4B, C). Concurrently, analysis of data from GSE138460 reveals that PROX1 expression is elevated in NEPC compared to CRPC (Fig. 4D). Correspondingly, Chromatin Accessibility data demonstrated an enhanced open chromatin configuration specifically at the transcription start site (TSS) of PROX1 (Fig. 4E). These findings indicate that PROX1 contributes to the development of drug resistance. Moreover, a significantly elevated PROX1 footprint score was observed in LN-35 cells (Fig. 4F), suggesting its heightened functional role as an active transcription factor in the resistant state. Notably, we observed that SMARCA4 knockdown in LN-35 cells significantly reduced PROX1 mRNA expression levels (Fig. 4G). Direct binding was confirmed using ChIP-seq analysis, which revealed higher SMARCA4 binding peaks at the PROX1 locus in LN-35 cells (Fig. 4H). Furthermore, analysis of public databases revealed a positive correlation between SMARCA4 and PROX1 (Fig. 4I). To definitively validate the physical association, Co-Immunoprecipitation (Co-IP) assays were performed, which confirmed a robust interaction between the SMARCA4 and PROX1 proteins in LN-35 cells (Fig. 4J). This establishes that SMARCA4 not only transcriptionally regulates PROX1 but also physically associates with its protein product, suggesting they may function within the same complex or pathway. Finally, we assessed the downstream functional role of PROX1. PROX1 knockdown in LN-35 cells significantly impaired the proliferative capacity, as demonstrated by EdU and colony formation assays (Fig. 4K, L). Analogously, Transwell and wound healing assays indicated that the migratory and invasive abilities of the cells were also substantially impaired following PROX1 silencing (Fig. 4M, N).

Building upon the established role of SMARCA4 in promoting neuroendocrine transdifferentiation, we investigated whether PROX1 similarly influences this process. Human NEPC single-cell RNA sequencing data from GSE264573 and GSE246155 revealed the enrichment of PROX1 in NEPC (Supplementary Fig. 4A, B). Clinical relevance was supported by analysis of SU2C patient data, demonstrating that high PROX1 expression significantly correlates with a propensity to develop neuroendocrine features (Fig. 4O). Functionally, PROX1 knockdown in LN-35 cells directly impacted the NE phenotype, leading to a decrease in both protein and mRNA levels of neuroendocrine markers (Fig. 4P, Q). Further dynamic evidence was gathered using our engineered ASCL1 and OCT4 fluorescence knock-in cells, where PROX1 silencing resulted in a progressively diminished fluorescence intensity over time (Fig. 4R), confirming its necessity for maintaining the neuroendocrine transdifferentiation. Furthermore, sphere formation assays showed that PROX1 knockdown significantly reduced the sphere-forming capacity (Fig. 4S), indicating its crucial role in maintaining cancer stemness associated with NET. In summary, these data establish that the aberrant activation of the chromatin remodeler SMARCA4 promotes malignant tumor progression and neuroendocrine transformation by directly binding to and upregulating the expression of the critical effector PROX1.

### SMARCA4 facilitates tumor progression through augmenting PROX1 histone acetylation

**Fig. 5:**
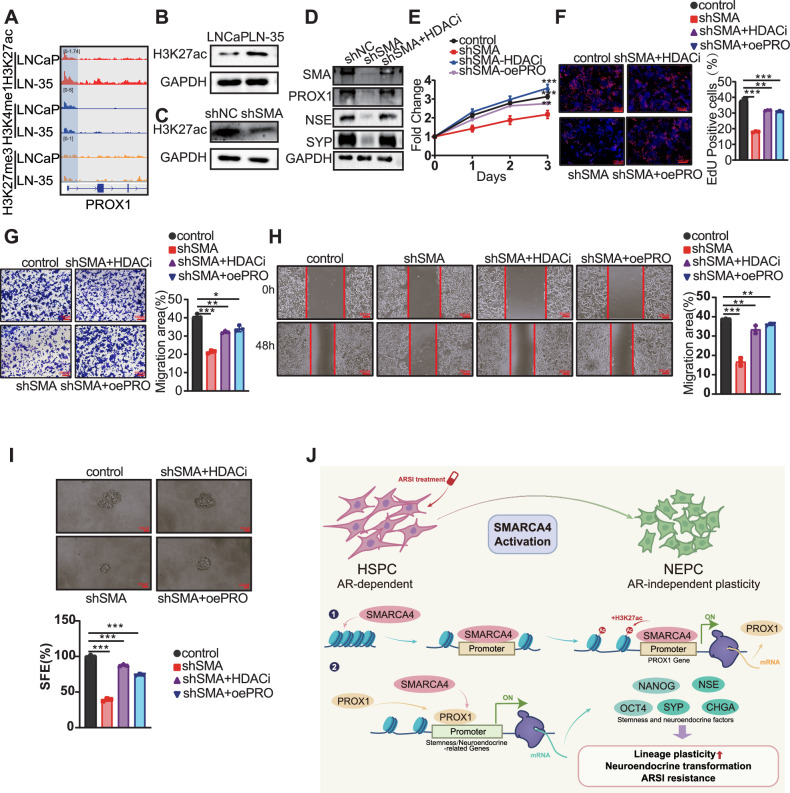
SMARCA4 promotes tumor progression by enhancing histone acetylation at the PROX1 locus. **A** Genome browser tracks showing histone modifications at the PROX1 locus in LNCaP and LN-35 cells. **B** Western blot analysis of global H3K27ac levels in LNCaP cells and LNCaP-35 cells. **C** Western blot analysis of global H3K27ac levels in LNCaP-35 cells following SMARCA4 knockdown. **D** Western blot analysis of PROX1 and NE markers in resistant cells subjected to SMARCA4 knockdown alone or in combination with the HDAC inhibitor entinostat. **E** Proliferation kinetics of LN-35 cells assessed by CCK-8 assay over a 4-day course (Days 0–3). Experimental conditions included: control, SMARCA4 knockdown, SMARCA4 knockdown combined with HDACi, and SMARCA4 knockdown combined with PROX1 overexpression. **F** Representative EdU staining images (left) and quantification of the percentage of EdU-positive cells (right) in LN-35 cells under the indicated conditions. **G** Representative images of Transwell assays (left) and quantification of the relative number of migrated cells (right) under the indicated conditions. Data are normalized to the control group (set as 100%). **H** Wound healing assay of LN-35 cells under the indicated conditions. Left: Representative images acquired at 0 and 48 hours. Right: Quantification of the wound closure rate, calculated as the ratio of migration distance at 48 hours to the initial wound width. **I** Representative images (left) and quantification (right) of tumor sphere formation in LN-35 cells under the indicated conditions. **J** Schematic illustration of the proposed working model depicting the role of SMARCA4 in driving enzalutamide resistance. (*p < 0.05, **p < 0.01, ***p < 0.001).

To delineate the mechanism by which SMARCA4 promotes PROX1 expression and mediates its oncogenic function, we examined histone modification patterns at the PROX1 locus. This analysis identified significant differences in the active histone mark H3K27ac (Fig. 5A). Western blot results similarly showed that H3K27ac levels were higher in LN-35 cells compared to LNCaP cells (Fig. 5B). Crucially, SMARCA4 knockdown in LN-35 cells resulted in a reduction in H3K27ac levels (Fig. 5C). This evidence suggests that SMARCA4 enhances H3K27ac modification, thereby promoting PROX1 transcription, which ultimately drives tumor progression. To validate this epigenetic mechanism, we examined the effect of the histone deacetylase inhibitor (HDACi) entinostat. Western blot analysis showed that while SMARCA4 knockdown reduced the expression levels of both PROX1 and neuroendocrine markers, the subsequent addition of HDACi effectively reversed this effect (Fig. 5D). These findings confirm that SMARCA4 promotes PROX1 expression by enhancing the active histone mark H3K27ac at its locus. To functionally validate this result, we performed rescue experiments focusing on key oncogenic phenotypes. SMARCA4 knockdown significantly suppressed cellular proliferation in LN-35 cells, as measured by CCK-8 and EdU assays. While HDACi treatment restored the proliferative ability of LN-35-shSMARCA4 cells. Notably, PROX1 overexpression following SMARCA4 knockdown similarly rescued the proliferative capacity, achieving restoration levels comparable to those achieved with HDACi treatment (Fig. 5E, F). Transwell and Wound Healing assays demonstrated that the impaired migratory and invasive capabilities resulting from SMARCA4 silencing were similarly restored by both HDACi treatment and PROX1 overexpression (Fig. 5G, H). Furthermore, Sphere Formation assays confirmed that both the HDACi and PROX1 overexpression could effectively rescue the cancer stemness properties suppressed by SMARCA4 knockdown (Fig. 5I). These results collectively demonstrate that SMARCA4 knockdown suppresses PROX1 expression and function by reducing H3K27ac modification at the PROX1 locus.

## Discussion

Despite the foundational role of androgen receptor (AR) targeting in prostate cancer therapy, the widespread use of second-generation androgen deprivation therapy (ADT) is frequently compromised by resistance driven by lineage plasticity, specifically the transition to neuroendocrine prostate cancer (NEPC) [25–28]. Our research elucidates this resistance evolution as a dynamic, multi-stage process—progressing from androgen dependence through early resistance mechanisms to ultimately activate neuroendocrine-related pathways. Focusing on this neuroendocrine transformation stage, we identified the chromatin remodeler SMARCA4 as a core driver of lineage plasticity. Mechanistically, SMARCA4 enhances this plasticity and confers NE characteristics by increasing H3K27ac levels of PROX1 to facilitate its chromatin accessibility, thereby upregulating the oncogenic effector PROX1 (Fig. 5J). Functionally, both in vitro and in vivo experiments demonstrated that SMARCA4 knockdown effectively suppresses malignant tumor progression and blocks the neuroendocrine transformation process. Collectively, our findings establish the SMARCA4/H3K27ac/PROX1 axis as a central regulatory mechanism underlying enzalutamide resistance and malignant progression, validating SMARCA4 as a highly promising therapeutic target for treating NEPC-driven refractory prostate tumors.

We simulated the process of resistance by constructing gradient-resistant cell lines and subjecting them to integrated multi-omics analysis. Firstly, transcriptome analysis revealed dynamic changes in cells during enzalutamide resistance, where the essential transcription factors varied across different stages. However, neuroendocrine (NE) alterations persisted throughout the entire resistance process, with a significant enrichment of the NE pathway, particularly in the terminal stage. This aligns with previous literature suggesting that prostate cancer cells undergo NE transdifferentiation under drug selection pressure, due to the epigenomic reprogramming of the pioneer factor FOXA1 [29]. Concurrently, chromatin remodeling-associated transcription factors played a crucial role in the terminal resistance stage. Changes in chromatin accessibility mirrored the transcriptome shifts: the expression of AR-axis signature genes and their corresponding chromatin openness were both reduced in resistant cells, while the expression of NE and stemness signaling pathways increased alongside enhanced chromatin accessibility peaks. This indicates that alterations in chromatin accessibility correspond to the overall trend of changes in resistant cells. Consistent with existing research, prior literature demonstrated that activated PAX6 signaling reprograms chromatin accessibility via the MET/STAT5A axis, thereby enhancing lineage plasticity and conferring a NE phenotype to tumors, ultimately resulting in treatment resistance in prostate cancer [30]. Furthermore, Zeb1 has been shown to upregulate histone lactylation signals, leading to enhanced corresponding chromatin accessibility, thus promoting lineage plasticity and NE differentiation in prostate cancer cells [31].

Our investigation revealed that the chromatin remodeler SMARCA4 is significantly upregulated during enzalutamide treatment-induced neuroendocrine transformation. Both Pathway enrichment analysis and IPA results consistently highlighted SMARCA4’s critical role in this process. Supporting these cellular findings, analysis of public databases further validated the association between higher SMARCA4 expression and poor patient prognosis in NEPC. Moreover, IGV visualization demonstrated specific alterations at the SMARCA4 locus, including increased chromatin accessibility and reduced repressive H3K27me3 modification. These epigenetic alterations align perfectly with the overall adaptive trajectory observed during the development of drug resistance. Furthermore, comprehensive in vitro and in vivo experiments confirmed that aberrant SMARCA4 activation promotes malignant tumor progression and facilitates neuroendocrine transformation in prostate cancer. While SMARCA4 is clearly correlated with neuroendocrine tumors, its functional role is context-dependent across different cancer types. For instance, in small cell lung cancer, SMARCA4 promotes tumor progression by directly binding to and regulating classical neuroendocrine markers such as ASCL1 to maintain the NE state [32]. Conversely, in gastrointestinal cancers, SMARCA4 can act as a tumor suppressor, with reported SMARCA4-deficient cases showing positive immunohistochemical staining for NE markers like Syn, CgA, and CD56 [33]. Therefore, given the literature demonstrating SMARCA4’s dichotomous role, elucidating the precise lineage-specific mechanism of SMARCA4 in neuroendocrine prostate cancer holds significant clinical implications for developing targeted therapies against treatment-resistant disease.

To fully dissect the functional mechanism of SMARCA4 in NEPC, we employed integrated multi-omics analysis to screen for its potential regulatory targets and identified PROX1 as a critical downstream candidate mediating tumor progression. Our initial screen revealed that PROX1 exhibits elevated expression in drug-resistant cells, concurrent with progressively enhanced chromatin accessibility at its locus. Consistent with its known function as a transcription factor, footprinting analysis confirmed this heightened activity by showing progressively increasing PROX1 footprint scores in resistant cells. Furthermore, in vitro experiments validated that PROX1 actively promotes malignant progression and neuroendocrine transformation, strongly suggesting its essential role in mediating acquired drug resistance. Crucially, protein-protein interaction studies and other relevant experiments established that PROX1 functions as a direct downstream target regulated by SMARCA4. Then we determined that SMARCA4 enhances the active histone mark H3K27ac modification at the PROX1 locus, which subsequently increases chromatin accessibility and ultimately promotes the observed lineage plasticity. Existing studies have established PROX1’s involvement in various malignancies, such as glioma, where PROX1 loss is reported to promote the transition of proneural cells to the aggressive mesenchymal subtype [34]. Furthermore, other work suggests that PROX1 downregulation facilitates non-canonical reprogramming [35]. These findings present an apparent contrast to our results, which propose that the aberrant activation of PROX1 enhances lineage plasticity in enzalutamide-resistant prostate cancer. However, considering the vast tumor heterogeneity of transcription factors, this apparent discrepancy likely reflects the functional complexity of PROX1 across different cellular and malignant environments. Our mechanistic findings align with known epigenetic regulatory mechanisms. It has been shown that the SWI/SNF complex (of which SMARCA4 is a core component) can interact with P300 to enhance H3K27ac levels at downstream genes, thereby facilitating the complex’s binding to target gene enhancers [36]. Consistent with this framework, our study reveals that SMARCA4 elevates H3K27ac modification at the PROX1 locus, providing the epigenetic mechanism by which SMARCA4 confers neuroendocrine transformation capability to drug-resistant prostate cancer cells.

Notably, although our study establishes that SMARCA4 activates PROX1 transcription via H3K27ac enrichment, the specific histone acetyltransferase (HAT) responsible for this acetylation remains undefined. While SMARCA4 is known to function in concert with HATs like p300, direct evidence in this specific context is currently lacking. Therefore, identifying SMARCA4 co-factors—potentially through p300/CBP inhibition or protein-protein interaction assays—will be crucial for comprehensively mapping this epigenetic network and represents a primary focus of our future research. The core findings are derived from established drug-resistant cell lines and in vivo mouse models, which allowed us to model the development of enzalutamide resistance. However, a significant limitation is the lack of validation using human clinical specimens. Future work must prioritize the acquisition and analysis of human tissue samples from patients progressing to neuroendocrine prostate cancer (NEPC) to confirm the clinical relevance and generalizability of the SMARCA4 pathway in human disease progression. Drug resistance is a dynamic and evolving process. Our findings clearly delineate SMARCA4’s critical role in the advanced stages of resistance under high drug concentration conditions. However, the specific transcription factors and molecular mechanisms that govern the transition during the early and intermediate resistance phases remain to be fully characterized. A comprehensive temporal analysis to identify these initial drivers will constitute a primary focus for our subsequent investigations.

This study investigated the mechanisms driving enzalutamide resistance using a gradient-resistant cell model series. Our results demonstrated that resistant cells undergo lineage plasticity, characterized by neuroendocrine alterations that persist throughout the resistance progression. Critically, these phenotypic changes were underpinned by epigenetic reprogramming. Of note, we identified the chromatin remodeler SMARCA4 as a central factor; it upregulates the active histone mark H3K27ac at the PROX1 locus. Functionally, this SMARCA4/H3K27ac/PROX1 axis drives malignant progression and confers the neuroendocrine phenotype. Collectively, these findings establish the epigenetic axis driven by SMARCA4 as a crucial vulnerability in therapy-resistant disease, indicating that targeting SMARCA4 represents a promising therapeutic strategy for patients who have developed resistance following enzalutamide treatment.

## Materials and Methods

### Cell culture and establishment

The LNCaP cell line was purchased from Procell Life Science & Technology Co., Ltd. (Wuhan, China). Prior to use, all cell lines were routinely authenticated by STR profiling and screened to be mycoplasma-negative. Cells were maintained at 37 °C in a CO₂ incubator using RPMI-1640 Complete Medium (Procell) supplemented with 1% penicillin/streptomycin (Thermo Fisher Scientific, Massachusetts, USA). To establish enzalutamide-resistant sublines, LNCaP cells were first adapted to grow in RPMI-1640 medium containing charcoal-stripped fetal bovine serum (CS-FBS) to simulate an androgen-deprived environment. Resistance was then induced using a stepwise dose escalation protocol. Treatment began with 5 μM enzalutamide (MedChemExpress, Shanghai, China). After stable growth and passaging for 10 generations at this concentration, the enzalutamide concentration was sequentially increased to 10 μM and continued until the cells demonstrated stable proliferation at a final concentration of 35 μM enzalutamide.

### CCK8

To assess the level of drug resistance, cells were seeded in 96-well plates at a density of 1 × 10^4 cells/well. After 24 hours of adherence, the medium was replaced with culture medium containing various gradient concentrations of enzalutamide. The cells were then cultured for an additional seven days without medium replacement to ensure chronic drug exposure. Cell viability was measured using the CCK-8 assay (APExBIO, K1018, Shenzhen, China). The absorbance at 450 nm was subsequently measured using a microplate reader to calculate the IC₅₀ values.

To assess differences in cell proliferation capacity, cells subjected to various treatments were seeded into four separate 96-well plates (at a density of 1 × 10^4 cells/well. These plates were sequentially analyzed at 24-hour intervals, designated as Day 0, Day 1, Day 2, and Day 3. Cell viability for each plate was determined using the CCK-8 assay. Following the manufacturer’s instructions, 10 μL of CCK-8 reagent was added to the designated plate, and the cells were incubated at 37 °C for 2 hours. The absorbance at 450 nm was then measured using a microplate reader. The resulting absorbance values were used to plot a cell growth curve (line graph) illustrating proliferation over the four-day period.

### EdU proliferation assay

The cell proliferation assay utilized the EdU Flow Cytometry Assay Kit (APExBIO, K1078). Initially, 1 × 10⁴ cells were seeded into individual wells of a 96-well plate. After a 48-hour incubation period, the cells were treated by adding an appropriate volume of 20 μM 2× EdU working solution to the culture medium, followed by a 2-hour incubation at 37 °C. Following the manufacturer’s protocol, the cells were subjected to the Click reaction mixture for 30 minutes. After washing with PBS, cells were counterstained with Hoechst 33342 for 15 minutes to visualize the nuclei. Finally, the stained cells were analyzed using a fluorescence microscope, and the density of EdU-positive cells (indicating proliferating cells) was quantified using ImageJ software.

### Colony formation assay

Cells were seeded at a low density of 1 × 10³ cells per well in 6-well plates to allow for colony formation. The cells were maintained in culture with the medium refreshed every three days. Following a two-week incubation period, the culture medium was aspirated, and gently washed with Phosphate-Buffered Saline (PBS). The colonies were then fixed using 4% paraformaldehyde for 15 minutes and subsequently stained for 20 minutes with 0.1% crystal violet. Finally, the density of the colony-forming cells was quantitatively assessed using ImageJ software.

### ATAC-seq

Cells (1 × 10⁵ cells) were collected and pelleted by centrifugation at 2300 rpm for 5 minutes at 4 °C. Sample preparation was subsequently performed using the Hyperactive ATAC-Seq Library Prep Kit for Illumina (Vazyme, TD711, Nanjing, China). Following lysis, the cells were subjected to the tagmentation reaction using the MIX reaction cocktail from the kit. The resulting fragmented DNA products were isolated via ATAC DNA Extract Beads. Library amplification was then carried out, utilizing the manufacturer’s recommended Library Amplification Reaction Mix, typically consisting of 12 amplification cycles. The amplified libraries were purified using ATAC DNA Clean Beads, and their concentration was precisely determined using the Equalbit 1× dsDNA HS (High Sensitivity) Assay Kit (Vazyme, EQ121). Only qualified libraries proceeded to the downstream sequencing process.

### ChIP-Seq

Sample preparation was performed using the SimpleChIP® Enzymatic Chromatin IP Kit (Cell Signaling Technology, 9003S, Massachusetts, USA). Initially, cells were fixed with formaldehyde. A total of 1 × 10⁵ cells were then collected and pelleted by centrifugation at 600 × *g* for 5 minutes at 4 °C. Following the manufacturer’s protocol, the cells were lysed and treated with prepared buffers. Chromatin was then isolated and fragmented by disrupting nuclear membranes using a non-contact ultrasonic cell disruptor to achieve the desired fragment size. Specific antibodies were added to the fragmented chromatin samples and incubated overnight at 4 °C to immunoprecipitate the target protein-DNA complexes. The complexes were subsequently captured using Protein G Magnetic Beads, and the associated DNA was purified. Finally, the concentration of the purified DNA was quantified using a Qubit 4.0 Fluorometer, and only qualified samples proceeded to downstream sequencing.

### Cut& Tag

Sample preparation was executed using the Hieff NGS® In-Situ DNA Binding Profiling Library Prep Kit for Illumina® V2 (Yeasen, 12597ES, Shanghai, China), which is based on the CUT&Tag methodology. A total of 1 × 10⁵ cells were harvested and pelleted by centrifugation at 600 × *g* for 5 minutes at 4 °C. The cells were then resuspended in 1× Wash Buffer and incubated with ConA Beads for 10 minutes. Next, the target primary antibody was introduced and incubated overnight at 4 °C. Following this, the secondary antibody was added using the prepared secondary antibody buffer, with incubation proceeding at room temperature for 1 hour. Subsequently, Transposase was added and incubated at room temperature for 1 hour, after which it was activated by adding 1× Activating Buffer and incubating at 37 °C. The resulting DNA library was then amplified and purified according to the protocol, typically employing 12 amplification cycles. Finally, the DNA library concentration was precisely quantified using a Qubit 4.0 Fluorometer, and only qualified samples proceeded to downstream sequencing.

### Tumorsphere formation assay

To investigate tumor sphere formation, cells were resuspended in RPMI-1640 medium supplemented with 1:50 B27, 20 ng/mL epidermal growth factor, and 20 ng/mL basic fibroblast growth factor. Subsequently, the cells were plated at a density of 1000 cells per 100 μL into 96-well ultra-low attachment plates (Corning, 3474, New York, USA). The culture was maintained for 10 days, with the addition of 50 μL of fresh medium to each well every 3–4 days. Upon completion of the incubation period, the resulting tumor spheres were photographed under a microscope, and the number and size of the spheres in the different experimental groups were quantitatively analyzed.

### Tumor xenograft experiment

The experimental protocol received approval from the Experimental Animal Care Commission of Ruiye Model Animal Biotechnology Co., Ltd. (Guangzhou, China). Four-week-old male NSFG mice, sourced from Xuzhou Nuohang Institute of Biotechnology Co., Ltd. (Xuzhou, China) were housed at a density of five per cage and underwent a one-week acclimatization period prior to the start of the experiment, during which their health status was carefully monitored. Twelve mice were randomly assigned to either the LNCaP-35 group or the LNCaP-35-shSMARCA4 group, with six mice per group.

For the in vivo tumor growth experiment, LNCaP-35 and LNCaP-35-shSMARCA4 cells were collected and resuspended in a 1:1 mixture of PBS and high-concentration Matrigel (Corning, 354248). A 100 μL suspension (containing 1–2 × 10⁶ cells per injection) was injected into the left flank. Tumor volume was calculated using the formula: V = L × W² / 2. The day when the tumor volume reached 100 mm³ was designated as Day 0 for volume measurements.

### Western blotting

For protein extraction, cells were lysed using RIPA buffer supplemented with protease inhibitors, and total protein concentrations were quantified using the Pierce™ BCA Protein Assay Kit (Thermo Fisher Scientific, 23227). Samples were mixed with 5× loading buffer and denatured at 100 °C for 10 minutes. Subsequently, proteins were separated by 10% SDS-PAGE and transferred onto PVDF membranes. The membranes were blocked with 5% bovine serum albumin (BSA) in TBST for 1 hour at room temperature, followed by overnight incubation with primary antibodies at 4 °C. The primary antibodies used included: SMARCA4 (Proteintech, 21634-1-AP, 1:1000, Wuhan, China), PROX1 (Proteintech, 11067-2-AP, 1:5000), CHGA (Proteintech, 60135-2-Ig, 1:5000), NSE (Proteintech, 10149-1-AP, 1:5000), SYP (Proteintech, 17785-1-AP, 1:20000), and GAPDH (Santa Cruz Biotechnology, sc-47724, 1:1000, Texas, USA).

### Co-immunoprecipitation

Co-immunoprecipitation (Co-IP) assays were performed using Protein A/G Magnetic Beads (Selleck, B23201, Texas, USA) following the manufacturer’s instructions. Briefly, cells were harvested and lysed in buffer supplemented with protease inhibitors, followed by incubation on ice for 10 minutes. The lysates were clarified by centrifugation at 14,000 × *g* for 10 minutes at 4 °C. The resulting supernatants were then incubated with specific antibodies and magnetic beads to capture the target protein complexes. After washing the beads to remove non-specifically bound proteins, the immune complexes were eluted by adding 50 µL of 1× SDS-PAGE loading buffer and heating at 95 °C for 5 minutes. The supernatants (eluates) were collected for subsequent Western blot analysis.

### Quantitative real-time PCR

Cells were seeded in 12-well plates and, after 24 hours of adherence, subjected to the indicated treatments. Total RNA was isolated using the EZ-press RNA Purification Kit (EZBioscience, B0004D, Jiangsu, China) according to the manufacturer’s protocol. Subsequently, RNA was reverse-transcribed into cDNA using Hifair® Ⅲ 1st Strand cDNA Synthesis SuperMix (Yeasen, 11141ES60). Quantitative real-time PCR (qRT-PCR) was performed using qPCR SYBR Green Master Mix (Yeasen, 11201ES08). GAPDH served as the internal reference gene, and relative expression levels were calculated using the 2^−ΔΔCt^ method.

### Statistical analysis

All experiments in this study were performed with at least three independent replicates, and data are expressed as the mean ± standard error of the mean. Statistical comparisons between two groups were conducted using a two-tailed Student’s t-test, while comparisons among multiple groups were analyzed by one-way analysis of variance (ANOVA). All statistical analyses were performed using GraphPad Prism software (version 8.0.2 for Windows).

## Supplementary information

Supplementary MATERIAL

Western blot

## Data Availability

Public single-cell data were accessed via GSE264573 and GSE246155. For PROX1 mRNA expression and NCI-H660 cell line analysis, data were retrieved from GSE138460, while datasets for the correlation analysis of SMARCA4, PROX1, and neuroendocrine (NE) markers were sourced from GSE77930. The sequencing data generated in this study have been deposited in the Gene Expression Omnibus (GEO) database. The RNA-seq, ATAC-seq, and CUT&Tag data are accessible under accession numbers GSE318884, GSE318885, and GSE319070, respectively.
