# Supplementary material for: SMARCA4 promotes lineage plasticity and enzalutamide resistance in prostate cancer by regulating PROX1 via H3K27 acetylation
Source: Cell Death Discov. 2026 Mar 25;12:175. doi: 10.1038/s41420-026-03068-0 (PMC13039790; doi:10.1038/s41420-026-03068-0)
Supplement: Supplementary file 1 — Supplementary MATERIAL [file 41420_2026_3068_MOESM1_ESM.docx]

**SMARCA4 Promotes Lineage Plasticity and Enzalutamide Resistance in Prostate Cancer by Regulating PROX1 via H3K27 Acetylation**

Chenwei Wu^1,#^, Mayao Luo^2,#^, Chaojian Wu^1^, Yi Yuan^1^, Yadong Li^1^, Yuanpeng Liao^1^, Yifan Zhang^2^, Xin Huang^1,3^, Mengqi Wang^2^, Shidong Lv^2,3,*^, Qiang Wei^1,2,3,*^

^1^ Department of Urology, Nanfang Hospital, Southern Medical University, Guangzhou, Guangdong, 510515, China.

^2^ Department of Urology, Guangdong Cardiovascular Institute, Guangdong Provincial People's Hospital, Guangdong Academy of Medical Sciences, Southern Medical University, Guangzhou, Guangdong, 510080, China

^3^ Department of Urology, Ganzhou Hospital-Nanfang Hospital, Southern Medical University, Ganzhou, Jiangxi, 341000, China

^#^ These authors contributed equally to this work

*These authors are co-corresponding authors.

**Supplementary Figure 1**


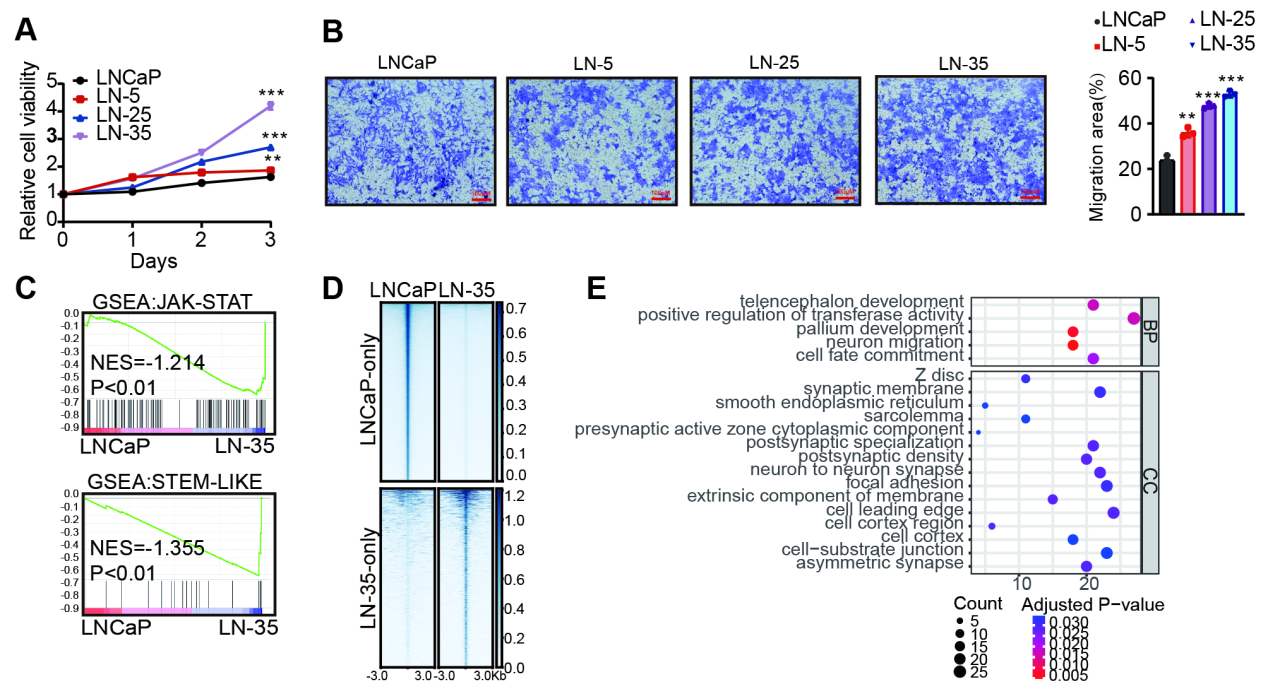


**Supplementary Figure 1.characterization of enzalutamide-resistant prostate cancer cell lines with varying resistance levels. (A)** Comparison of the proliferative capacity of LNCaP and resistant sublines. Cells were seeded at equal densities, and viability was assessed using the CCK-8 assay on Days 0, 1, 2, and 3. Data are presented as relative viability normalized to Day 0 (set as 100%). **(B)** Representative images of the Transwell migration assay and quantification of the percentage of area covered by migrated cells. **(C)** GSEA of transcriptomes from LNCaP versus LN-35 cells. **(D)** Heatmap visualization of ATAC-seq signal intensity across differentially accessible peaks in LNCaP versus LN-35 cells. **(E)** GO enrichment analysis of chromatin regions exhibiting increased accessibility in LN-35 cells. (*p < 0.05, **p < 0.01, ***p < 0.001).

**Supplementary Figure 2**





**Supplementary Figure 2. Epigenetic alterations in drug-resistant cells. (A)** Correlation analysis between transcriptomic changes and alterations in chromatin accessibility. **(B)** Heatmap of H3K4me1 and H3K27ac in LNCaP versus LN-35 cells. **(C)** Comparison of the global H3K27me3 level between LNCaP and LN-35 cells. **(D)** The protein levels of H3K27me3 in LNCaP cells and LN-35 cells.

**Supplementary Figure 3**





**Supplementary Figure 3. SMARCA4 promotes malignant phenotypes in prostate cancer. (A)** Proliferation kinetics of LN-35 cells following SMARCA4 knockdown, assessed by CCK-8 assay over a 4-day period (Days 0–3). Data are presented as fold change relative to Day 0. **(B)** Wound healing assay assessing the migration ability of LN-35 cells following SMARCA4 knockdown. Left: Representative images acquired at 0 and 48 hours. Right: Quantification of the wound closure rate, calculated as the ratio of migration distance at 48 hours to the initial wound width. **(C)** Flow cytometry analysis of apoptotic cells in control and SMARCA4-knockdown LN-35 cells. **(D)** Correlation analysis between SMARCA4 mRNA expression and NE gene in the GSE77930 dataset. **(E,F)** The enrichment of SMARCA4 and neuroendocrine marker expression across NEPC samples in the GSE264573 cohort. **(G)** The Expression of SMARCA4 across NEPC samples in the GSE246155 cohort. **(H,I)** mRNA (H) and protein (I) levels of NE markers in LN-35 cells following SMARCA4 knockdown. **(J)** Quantification of tumor sphere formation in control versus SMARCA4-knockdown LN-35 cells. **(K)** GO enrichment analysis of transcriptomic alterations in SMARCA4-knockdown LN-35 cells. (*p < 0.05, **p < 0.01, ***p < 0.001).

**Supplementary Figure 4.**

**

**

**Supplementary Figure 4. The Expression of PROX1 across NEPC samples.**

**(A,B)** The Expression of PROX1 across NEPC samples in the GSE264573 cohort (A) and in the GSE246155 cohort (B).
