# Supplementary material for: SMARCA4 promotes lineage plasticity and enzalutamide resistance in prostate cancer by regulating PROX1 via H3K27 acetylation
Source: Cell Death Discov. 2026 Mar 25;12:175. doi: 10.1038/s41420-026-03068-0 (PMC13039790; doi:10.1038/s41420-026-03068-0)

SMARCA4 Promotes Lineage Plasticity and Enzalutamide Resistance in Prostate Cancer by Regulating PROX1 via H3K27 Acetylation

Chenwei Wu[1,*], Mayao Luo[2,*], Chaojian Wu[1], Yi Yuan[1], Mengqi Wang[2], Yadong Li[1], Yuanpeng Liao[1], Shidong Lv[2,#], Qiang Wei[1,2,3,#]

[1] Department of Urology, Nanfang Hospital, Southern Medical University, Guangzhou, Guangdong 510515, China.

[2] Department of Urology, Guangdong Cardiovascular Institute, Guangdong Provincial People's Hospital, Guangdong Academy of Medical Sciences, Southern Medical University, Guangzhou, Guangdong, 510080, China

[3] Department of Urology, Ganzhou Hospital-Nanfang Hospital, Southern Medical University, Ganzhou, Jiangxi, 341000, China

Figure.1

Figure.1J

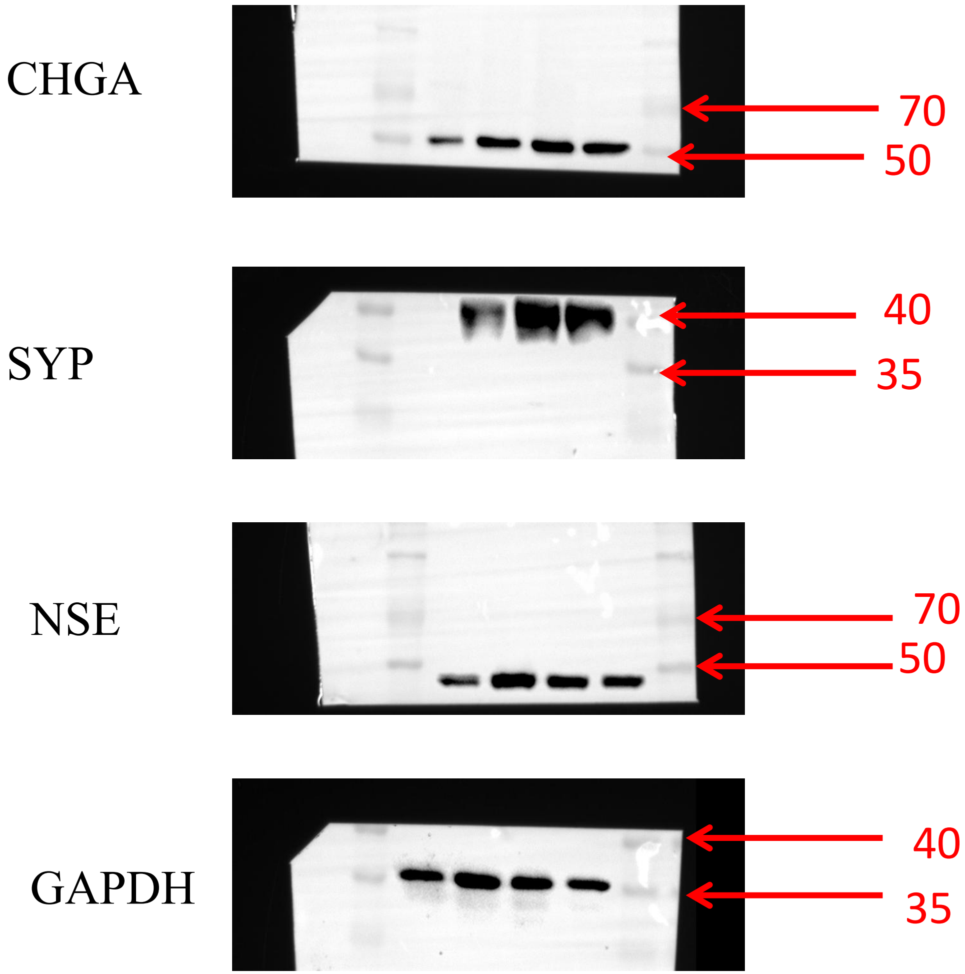

Supplemental Figure.2

Supplemental Figure.2B

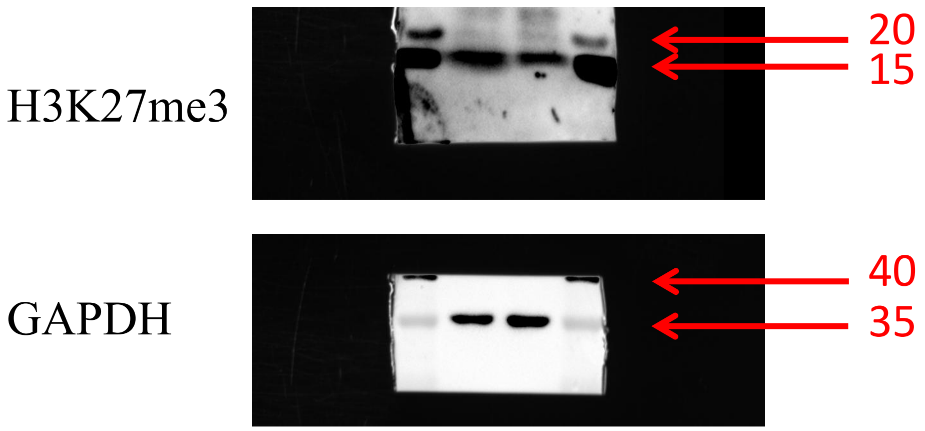

Figure.3

Figure.3E

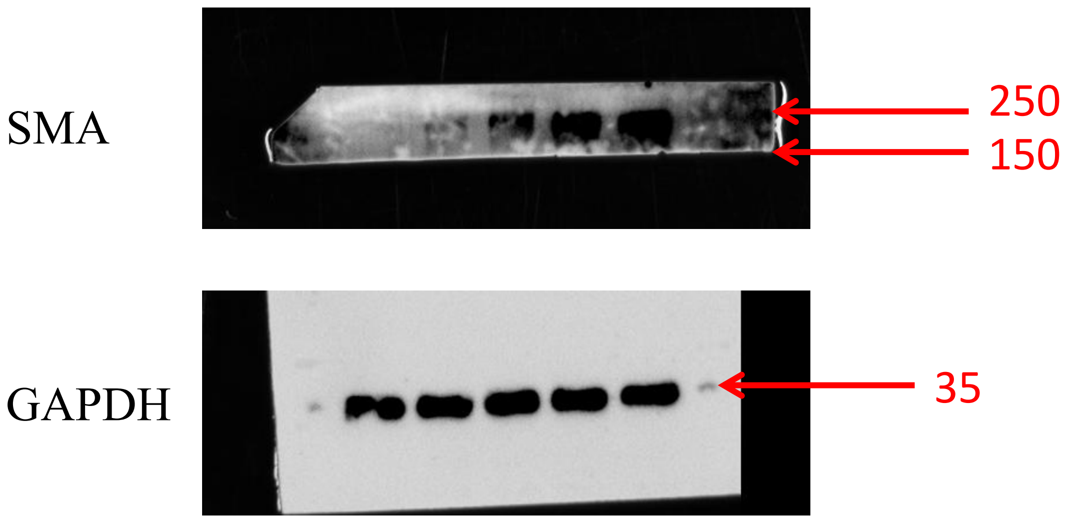

Figure.3O

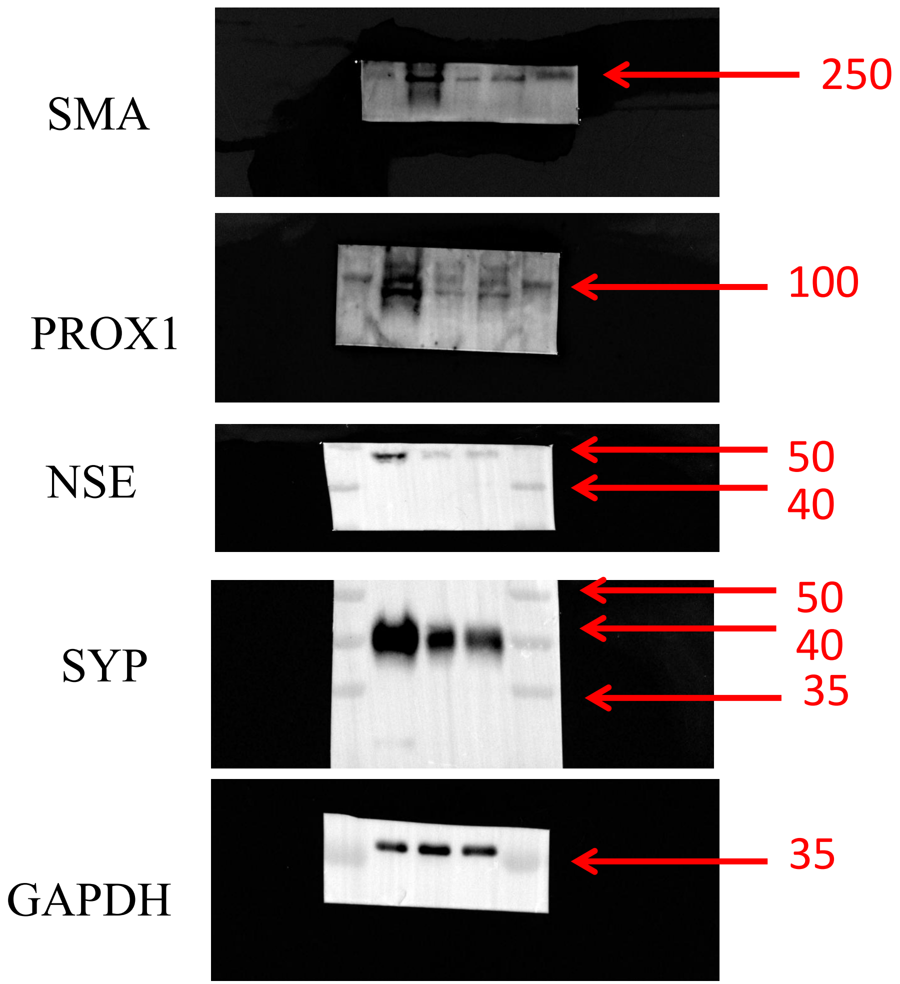

Supplemental Figure.3

Supplemental Figure.3G

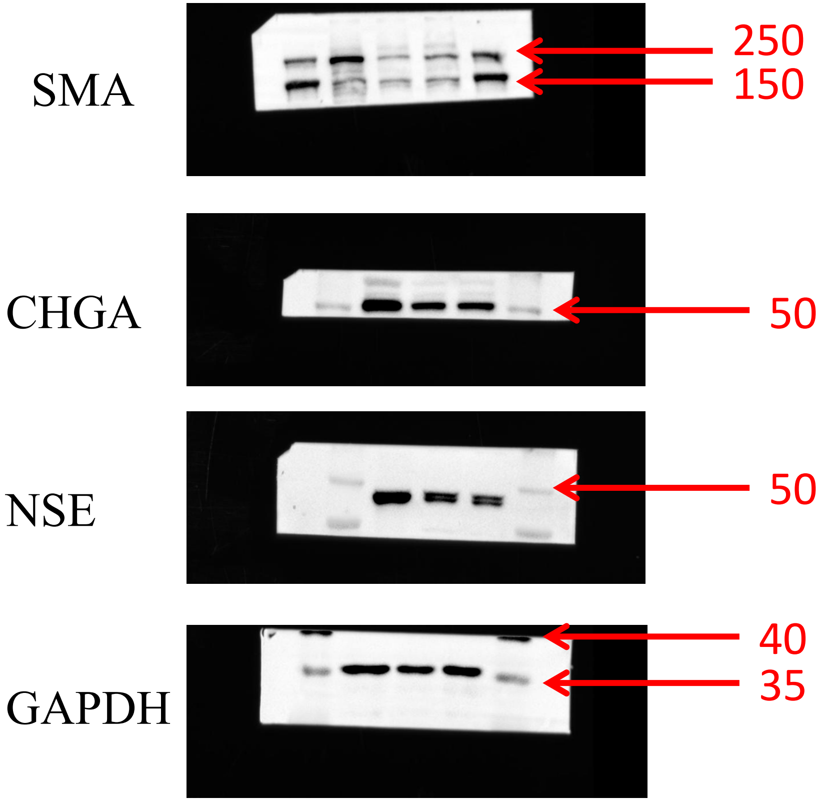

Figure.4

Figure.4B

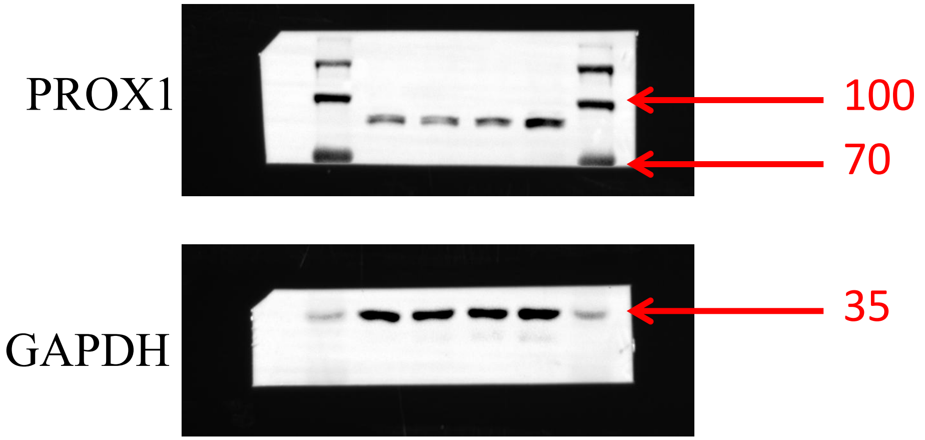

Figure.4J

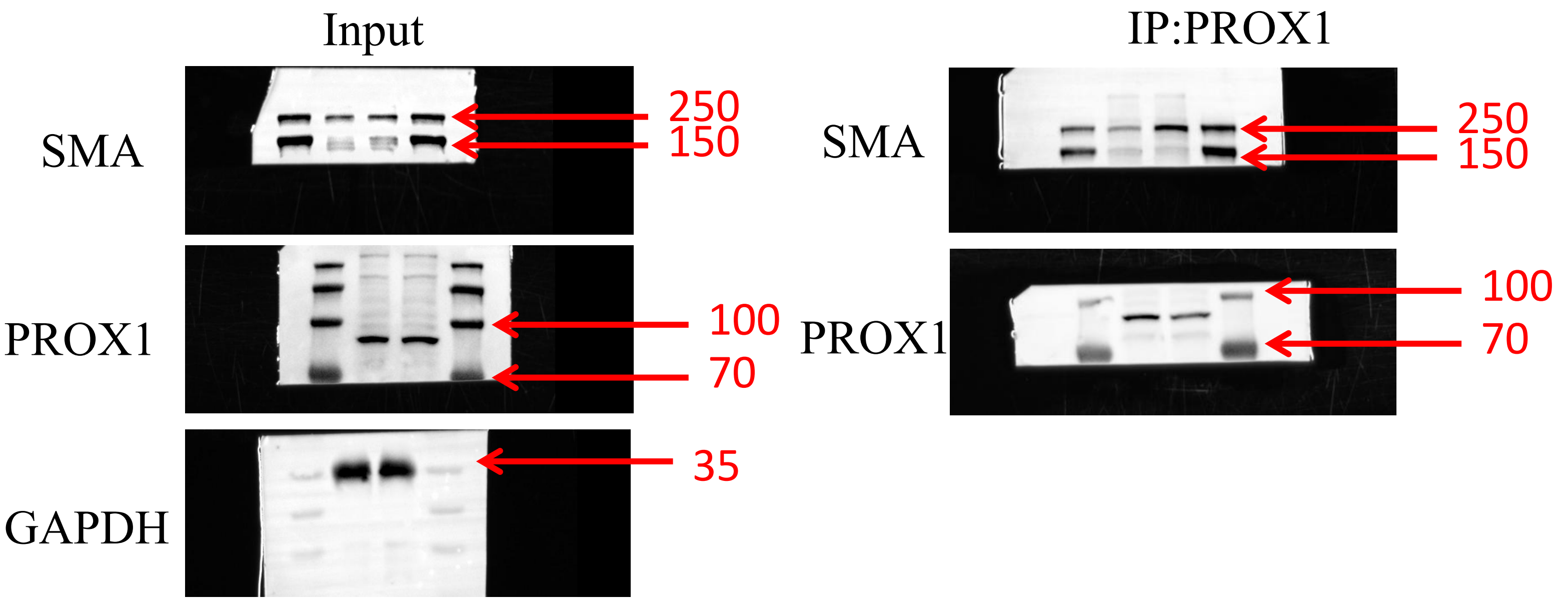

Figure.4P

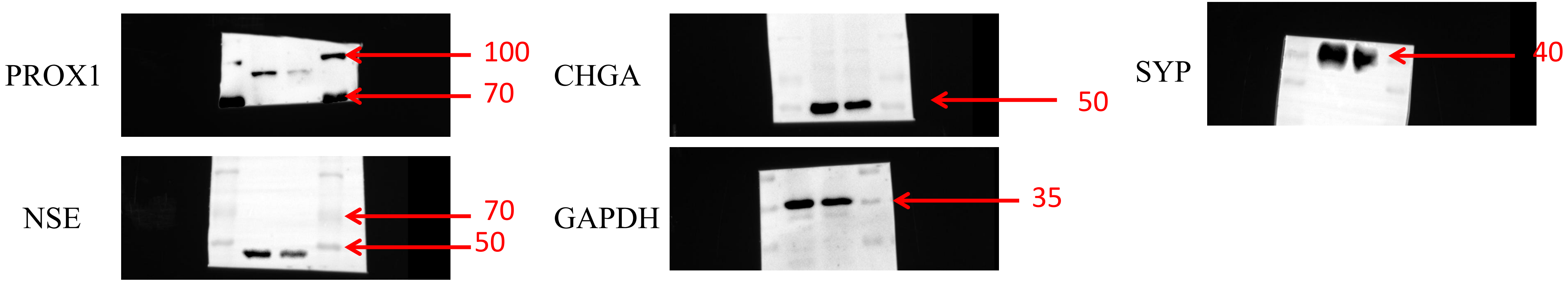

Figure.5

Figure.5B

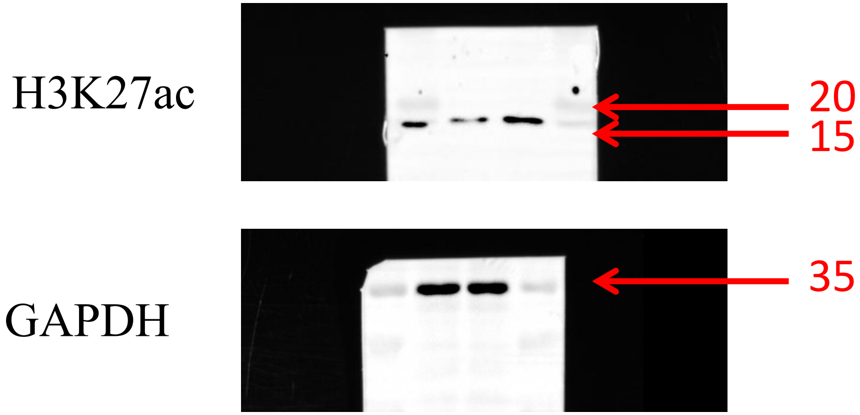

Figure.5C

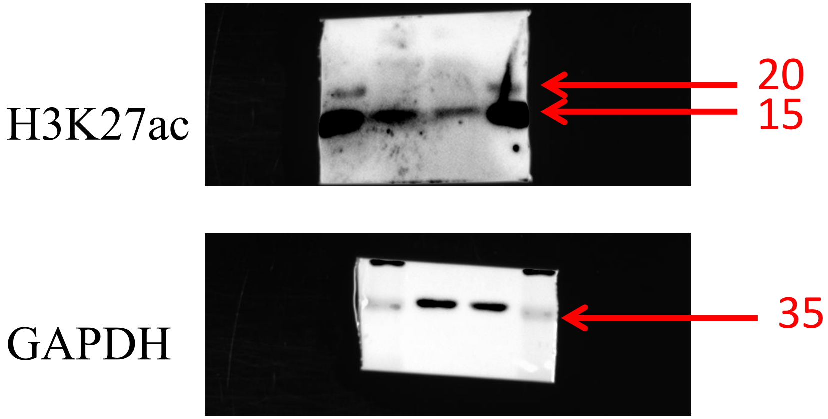

Figure.5D

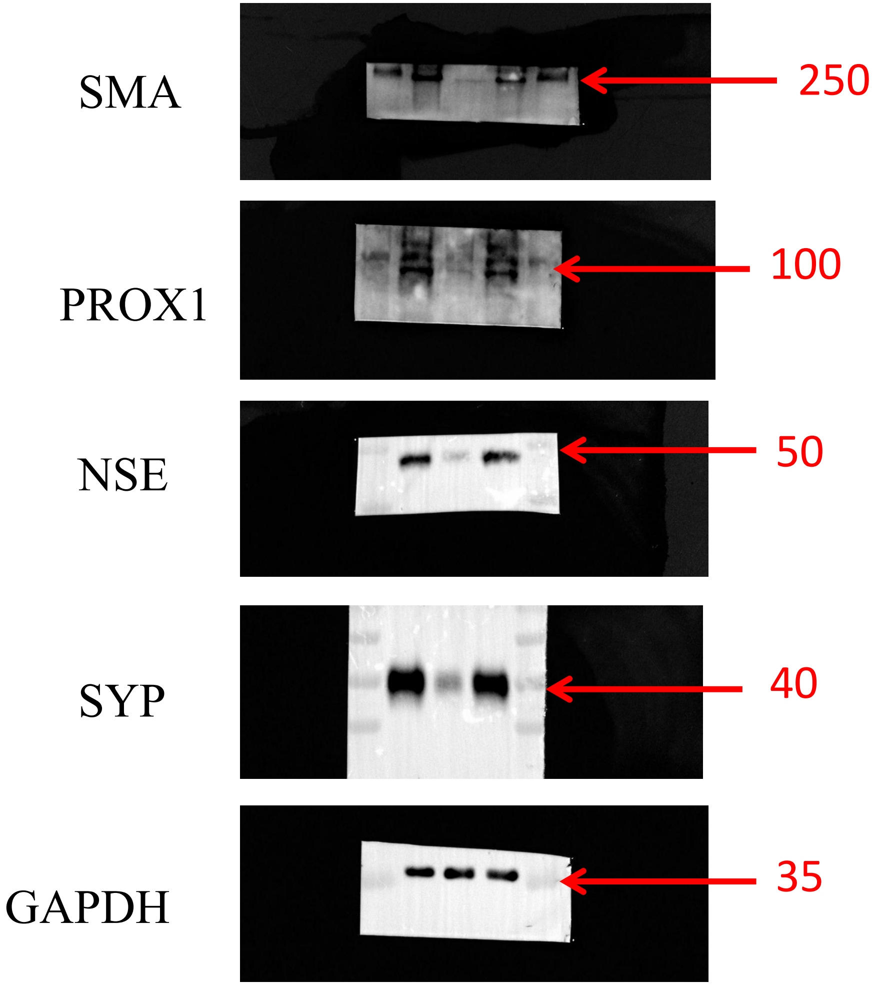

Supplement: Supplementary file 2 — Western blot [file 41420_2026_3068_MOESM2_ESM.pdf]
